# The R.B.N.A. and the College

**Published:** 1917-11-24

**Authors:** Isabel MacDonald

**Affiliations:** Secretary, Royal British Nurses' Association. 10 Orchard Street, W. 1


### THE EDITOR'S LETTER-BOX.

### The R.B.N.A. and the College.

We have received the following letter for publication :

The Secretary, the College of Nursing, Ltd.,
6 Vere Street, W. 1.

Dear Madam,—I have to acknowledge the receipt of your letter, which has been carefully considered by my executive committee.

The executive committee desire to state most emphatically that they do not for one moment admit that the proceedings with the Privy Council have been conducted in a dilatory manner. Any difficulties and delays which have arisen have been unavoidable so far as the Association is concerned, a statement which the Council will be prepared to prove at the proper time. I am to add that, although the powers granted under the Charter possessed by the Association are wide and far-reaching, the Supplemental Charter was admittedly intended to extend those powers. My Council, however, are advised that the alterations in the Supplemental Charter modify the document in such a substantial sense that practically no further powers are granted beyond those already possessed under the original Charter, except the power to change the name of the Association.

With reference to your remark, " If, however, the terms of our agreement with your Association are fulfilled, the College is prepared to take the further steps necessary to give effect to the amalgamation," my committee repudiate emphatically the unworthy insinuation that this Corporation has not fulfilled its part of the agreement.

Your notice to my Council "to refrain from incurring further expenditure" is unnecessary and misleading to those who are unaware of the circumstance that no further expense can be incurred in connection with the Supplemental Charter, seeing that the Lords of the Council have declined to grant the Supplemental Charter as agreed upon.

The proposal for the amalgamation of the College with the Royal British Nurses' Association (not the amalgamation of the Royal British Nurses' Association with the College, as stated in your letter), whereby the College would gain the privilege and prestige of possessing a Royal Charter, was cordially welcomed by our members. The single object of the Association was to benefit the profession by bringing about a closer union of the nursing world, and we stipulated for no special privileges for our members in return for what we were to share freely with the members of your Association. It is a matter for regret that circumstances over which this Association had no control have prevented the completion of the amalgamation.

With reference to your letter of the 9th inst., I enclose* herewith the copy of a letter conveying to the Lords of the Council the decision of the Council of this Corporation with regard to the Supplemental Charter, and also a copy of the reply received from them.—I am, yours faithfully,

(Signed) Isabel Macdonald,
Secretary, Royal British Nurses' Association.

10 Orchard Street, W. 1, November 12, 1917.

* No enclosure was sent with this letter, nor has one been sent on a notification of this omission.—Ed. The Hospital.

